# Recent and early 20th century destabilization of the subpolar North Atlantic recorded in bivalves

**DOI:** 10.1126/sciadv.adw3468

**Published:** 2025-10-03

**Authors:** Beatriz Arellano-Nava, Timothy M. Lenton, Chris A. Boulton, Sarah Holmes, James Scourse, Paul G. Butler, David J. Reynolds, Tamara Trofimova, Pierre Poitevin, Alejandro Román-González, Paul R. Halloran

**Affiliations:** ^1^ Global Systems Institute and Geography Department, University of Exeter, Exeter, UK.; ^2^ Met Office, Exeter, UK.; ^3^ World Meteorological Organization, Geneva, Switzerland.; ^4^ Centre for Geography and Environmental Science, University of Exeter, Exeter, UK.; ^5^ Institut des Sciences de la Mer, Université du Québec à Rimouski, Rimouski, Canada.; ^6^ School of the Environment and Life Sciences, University of Portsmouth, Portsmouth, UK.

## Abstract

Climate change risks triggering abrupt weakening in two climatically important North Atlantic Ocean circulation elements, the subpolar gyre and the Atlantic Meridional Overturning Circulation (AMOC). Loss of AMOC stability has been inferred from slowing recovery of temperature and salinity fluctuations over time. However, observational datasets, constructed from records with sparse spatial and temporal coverage, may introduce substantial biases in stability indicators. Alternative records are therefore needed for reliable stability assessments. Here, using bivalve-derived environmental reconstructions, we show that the subpolar North Atlantic has experienced two destabilization episodes over the past ~150 years. The first preceded the rapid circulation changes associated with the 1920s North Atlantic regime shift, suggesting that a tipping point may have been crossed in the early 20th century. The second and stronger destabilization began around 1950 and continues to the present, supporting evidence of recent stability loss and suggesting that the region is moving toward a tipping point.

## Teaser

Clams reveal North Atlantic destabilization in the early 20th century and at present.

## INTRODUCTION

One of the most concerning aspects of the climate crisis is the possibility of abrupt and potentially irreversible changes in some components of the Earth system, known as “tipping elements” (*1*). These elements can undergo self-propelled reorganization once a critical threshold, or tipping point, is crossed (*2*). An abrupt transition in a tipping element would have considerable climate impacts on regional to global scales, with serious implications for ecosystems and human societies (*2*, *3*). Two key tipping elements involve circulation changes in the Atlantic Ocean: the Atlantic Meridional Overturning Circulation (AMOC) and the North Atlantic subpolar gyre (SPG) (*3*). Both play a crucial role in redistributing heat across the planet, thereby modulating global climate. In response to anthropogenic climate change, both systems are at risk of passing a tipping point, leading to the collapse of deep convection (*3*, *4*). This collapse would weaken northward heat transport, potentially triggering regional cooling in the North Atlantic, a more seasonal climate in Western Europe, more frequent extreme weather events, and shifts in global precipitation patterns (*3*–*5*). While an AMOC collapse would have major planetary-scale consequences (*3*), an abrupt weakening of the SPG would still have substantial impacts, particularly on the North Atlantic region and surrounding areas (*4*, *5*).

Despite considerable advances in our understanding of these tipping elements, uncertainties remain regarding their current state and their likelihood of crossing a tipping point. This is mainly due to the difficulty of modeling tipping point behavior using Earth system models (*3*, *6*). As an alternative, a widely used approach for anticipating an approaching tipping point involves identifying the generic symptoms of a phenomenon known as critical slowing down (*6*, *7*). As a tipping point nears, the balancing feedbacks that normally restore a system to its current state get weaker. This means that small perturbations cause larger deviations from equilibrium and slower recoveries, leading to more pronounced and longer-lasting fluctuations (*2*, *7*). When far from a tipping point, a system can quickly return to equilibrium after a disturbance; as the tipping point approaches, recovery slows and the system increasingly resembles its previous states over time (*2*, *7*). This behavior is considered a symptom of the declining stability or resilience that usually precedes a tipping point (*7*, *8*). Critical slowing down is typically detected by measuring temporal changes in lag-1 autocorrelation [AR(1)] and variance (*6*, *7*, *9*), with the restoring rate (λ) recently introduced as an additional indicator of how quickly the system returns to equilibrium (*10*). Increases in these metrics over time suggest a loss of stability (*7*, *8*), i.e., a weakening of restoring feedback mechanisms, which is an early warning signal for the potential approach of a tipping point (*6*, *8*).

By applying this method to temperature and salinity reanalysis datasets (*10*, *11*), as well as to a reconstruction of Atlantic Multidecadal Variability (AMV) from land-derived proxy records (*12*), recent studies have found a slowdown in variability across much of the North Atlantic, which has been attributed to the destabilization of the AMOC (*10*, *11*). However, concerns remain regarding the robustness of these findings (*13*–*15*). Detecting early warning signals on the basis of critical slowing down requires long, regularly spaced time series (*6*, *8*), characteristics that are uncommon in instrumental records, particularly in marine data, which rarely extend before the mid-20th century. Consequently, comprehensive reanalysis datasets and reconstructions often rely on statistical techniques such as aggregation and interpolation, which are designed to preserve average values, often at the expense of higher-order statistics like autocorrelation and variance (*13*, *16*). As a result, record construction methods can introduce biases in these metrics, potentially leading to artificial trends and reducing their reliability for stability assessments (*13*, *16*, *17*).

A further concern surrounds the ability of proposed AMOC sea surface temperature and salinity fingerprints to effectively isolate changes in AMOC strength (*18*, *19*). The extent of AMOC influence on surface temperature patterns is still debated (*18*–*20*), and evidence suggests that these relationships may not hold outside the calibration period (*18*). Changes in North Atlantic temperatures, salinities, and AMOC strength are also influenced by SPG dynamics (*21*–*24*), a tipping element that could shift rapidly without necessarily causing a large-scale AMOC transition (*3*, *4*). Palaeoceanographic evidence indicates that the North Atlantic has experienced rapid reorganizations (*25*) linked to SPG dynamics throughout the Holocene (*26*), with the most recent being the transition into the Little Ice Age (*27*). This may be part of a complex stability landscape for the AMOC, with multiple alternative configurations (*28*). Given these complexities, there is a need for long-term, high-resolution reconstructions of North Atlantic variability from within the ocean that can track circulation dynamics.

Here, we assess changes in stability in the subpolar North Atlantic over the past two centuries using a network of marine climate reconstructions derived from bivalve shells to determine whether the region could be moving toward a tipping point. Bivalves form annual shell increments, with properties such as width and isotopic composition reflecting marine climate variability (*29*). For instance, oxygen isotope ratios capture the influence of different water masses and changes in their properties (*29*, *30*). Shell increment widths, on the other hand, integrate the complex interplay of processes influencing primary and secondary production and its export to the seabed, including temperature, ocean circulation, and ecological dynamics (*29*). Long-term, annually resolved, and precisely dated records are constructed by cross-dating and overlapping data from bivalves that lived in different periods (*31*). These attributes make bivalve records the gold standard in marine environmental reconstructions (*29*, *31*) and uniquely suited for assessing marine climate stability (*32*). Previous work has demonstrated their potential to provide reliable early warning signals for tipping points (*32*), with the three longest existing records showing a decline in stability likely linked to the SPG before the transition into the Little Ice Age (*27*). Given that bivalves integrate multiple aspects of ocean dynamics, including variability linked to regional circulation dynamics (*33*–*35*), assessing the raw records directly, without isolating or reconstructing specific variables, offers an opportunity to detect stability changes while avoiding potential artifacts introduced by statistical preprocessing.

Using two stability indicators, AR(1) and the restoring rate (λ), we start our analyses by identifying episodes of potential loss of stability in bivalve records over the past two centuries, followed by an assessment of the robustness and significance of the trends for each episode across the records. We then evaluate the sensitivity of bivalve records to regional temperature variability to determine whether the observed changes are driven by basin-scale processes and to identify common regions of sensitivity among records exhibiting different degrees of stability loss. Our results reveal two episodes of significant destabilization: one before the 1920s circulation regime shift and another in recent decades, with the strongest signal observed in records most sensitive to SPG temperature variability.

## RESULTS

We compiled 25 bivalve-derived records from the northern North Atlantic shelves, which were considered suitable for measuring changes in lag-1 autocorrelation and the restoring rate (λ) (see Materials and Methods and table S1). This compilation includes increment-width, oxygen, and carbon isotope records from the North Sea (*36*–*39*), Irish Sea (*40*, *41*), Hebridean Sea (*42*–*44*), English Channel (*45*), Faroe Shelf (*46*), north and southwest Iceland (*33*, *47*–*49*), northern Norway (*50*), and Newfoundland (*35*, *51*), derived from the shells of two bivalve species: *Arctica islandica* and *Glycymeris glycymeris* (fig. S1).

First, we explored changes in AR(1) and λ across all records with sufficient data since 1800 to identify potential intervals of stability loss. These intervals correspond to periods where both AR(1) and λ increase over time. Theoretically, under stable conditions, AR(1) is typically close to zero and increases toward 1 as instability grows (*7*), while λ is negative and approaches zero from below as destabilization occurs (*10*). For each identified episode of potential stability loss, we assessed the robustness and significance of the trends in each indicator. Trends were quantified using the Kendall τ coefficient, which ranges from −1 (strictly decreasing) to 1 (strictly increasing), with positive values indicating increasing trends in AR(1) and λ, suggesting a loss of stability (*9*). Because long-term trends can influence autocorrelation, all records were detrended before computing stability indicators to avoid spurious trends (*9*). Given that detrending is performed using a bandwidth parameter and the metrics are computed over a sliding window, we evaluated the robustness of our results across different combinations of detrending bandwidth and sliding window size (*52*). Last, we assessed the statistical significance of observed positive trends against a null model of surrogate time series preserving the spectral properties of each record (see Materials and Methods) (*9*, *52*).

### Changes in stability over the past two centuries

**Fig. 1.**
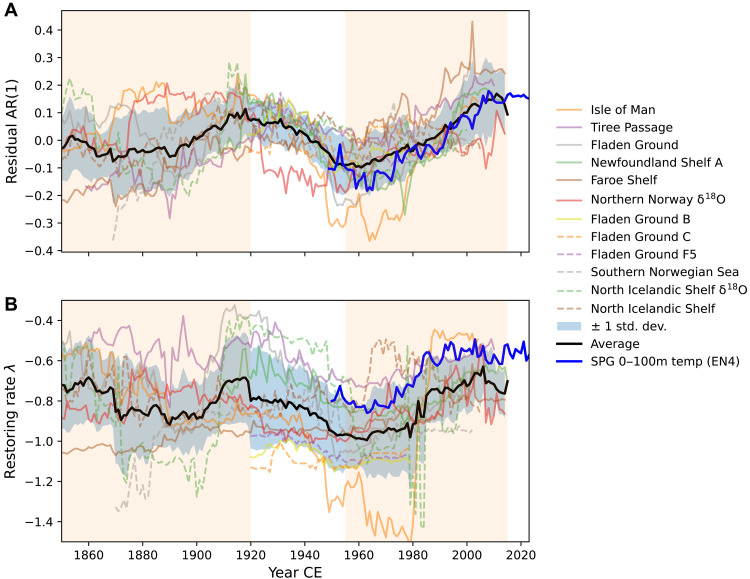
Multidecadal variability in AR(1) and the restoring rate λ. Temporal evolution of (**A**) AR(1) and (**B**) λ since the year 1800 from 12 records exhibiting a consistent pattern of variability. AR(1) and λ values were computed using a 50-year sliding window after detrending with a 15-year bandwidth. Values are plotted at the end of the sliding window. The black line shows the annually averaged values for each indicator across records, with the gray-blue–shaded area indicating one standard deviation around the mean. The thick blue line shows the temporal evolution of each indicator computed from SPG near-surface (0 to 100 m) EN4 temperature data. Orange-shaded intervals highlight periods showing potential stability loss.

An exploration of changes in AR(1) and λ across 22 sufficiently long records reveals a consistent pattern in 12 of them: Both AR(1) and λ exhibit high values around 1920, followed by a decline until approximately 1950, and a subsequent increase thereafter (Fig. 1). Consistency is stronger for AR(1) series (median *r* = 0.46, max = 0.99) than for λ (median *r* = 0.18, max = 0.99). Coherence increases when considering the period of 1920 onward, with median correlations of 0.60 for AR(1) and 0.40 for λ. For this analysis, AR(1) and λ values were computed using a 50-year sliding window moving 1 year at a time, with results assigned to the end of each window, so each data point reflects the state of the preceding five decades. This multidecadal pattern remains consistent when both AR(1) and λ are calculated using a larger window size (200 years) in sufficiently long records (fig. S2). Moreover, its presence in individual shell data (fig. S3) rules out the possibility of it being an artifact of record construction techniques.

To evaluate whether these intervals effectively exhibit significantly increasing trends in both indicators, we assessed robustness and significance in two periods: before and after 1920. This year was chosen because most of the records shown in Fig. 1 exhibit relatively high values for each indicator before this time, regardless of the window size used (Fig. 1 and fig. S2). These high values, which may indicate a transition between two regimes, would influence recent trends if not accounted for, so dividing the intervals around 1920 would minimize that influence and allows a clearer assessment of the potential loss of stability in each period. We present AR(1) results in the main text, with corresponding λ results provided in the Supplementary Materials.

**Fig. 2.**
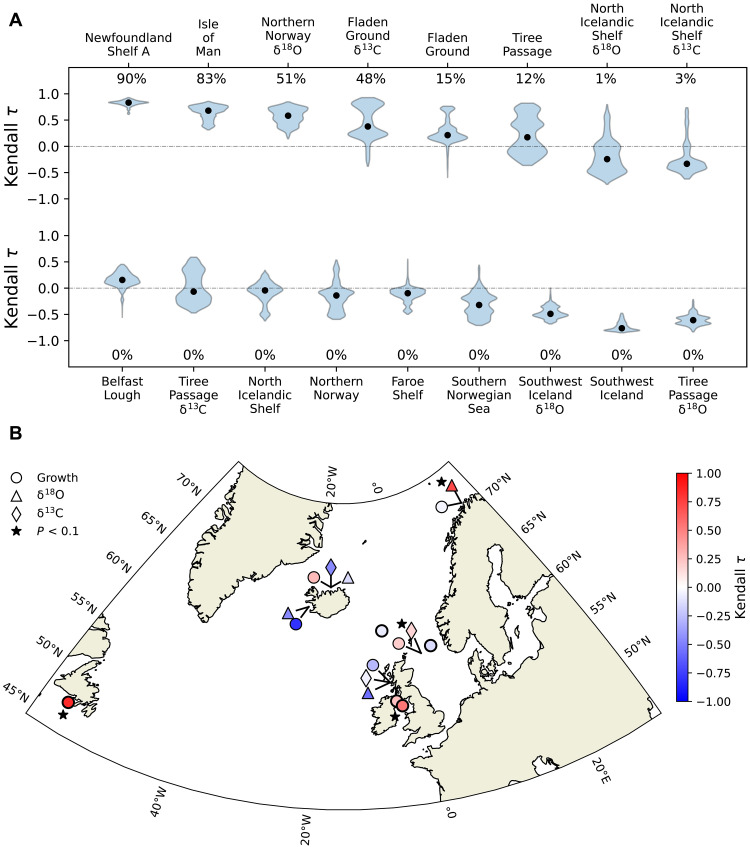
Robustness and significance of autocorrelation trends in bivalve records before 1920. (**A**) Distribution of Kendall τ trends in AR(1) for all combinations of sliding window and detrending bandwidth measured on each bivalve record between 1750 and 1920. Black circles represent the median Kendall τ values, while the percentages indicate the fraction of combinations exhibiting a significant trend. (**B**) Location of the records used to assess trends in AR(1) before 1920. The color of each marker represents the Kendall τ values after computing AR(1) using a 50-year sliding window and a 35-year detrending bandwidth. Redness is associated with the degree of loss of stability. Circles, triangles, and diamonds represent shell-growth, oxygen, and carbon isotope records, respectively. Records with at least 20% significant combinations are marked with a black star.

To enable the measurement of trends with large window sizes during the earliest interval, we selected all records with sufficient data between 1750 and 1920. To test the robustness of these trends to parameter choice, we assessed them across different combinations of sliding window size (ranging from 35 years to 80% of each record’s length) and detrending bandwidth (see Materials and Methods). Among the 17 selected records, six exhibit robust positive AR(1) trends (Fig. 2), with median Kendall τ values ranging from 0.25 to 0.83 (Fig. 2A). Of these, five series show significant trends (*P* < 0.1) in 15 to 90% of the tested parameter combinations. Some records, however, display noisy trajectories or persistently high AR(1) and λ values since 1850 (Fig. 1), resulting in weak or inconsistent trends between indicators. Despite this variability, the presence of significant trends suggests a regional loss of stability in the marine climate before the 1920s.

**Fig. 3.**
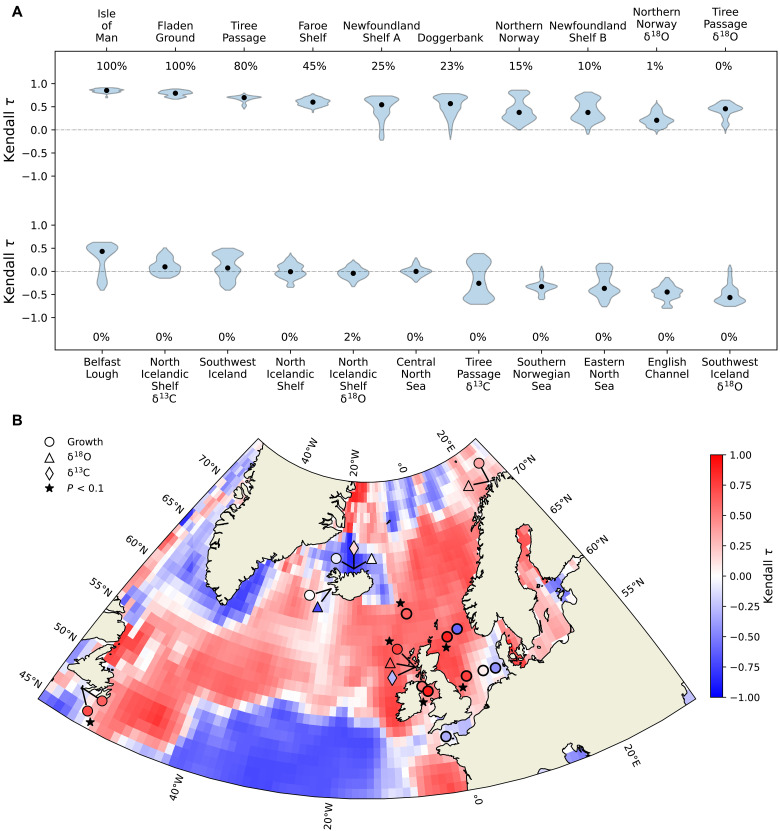
Robustness and significance of autocorrelation trends in bivalve records since 1920. (**A**) Distribution of Kendall τ trends in AR(1) for all combinations of sliding window and detrending bandwidth measured on each bivalve record since 1920. Black circles represent the median Kendall τ values, while the percentages indicate the fraction of combinations showing a significant trend. (**B**) Spatial trends in AR(1) measured on bivalve-derived records and northern North Atlantic sea surface temperatures. The scattered figures represent AR(1) trends measured on bivalve records since 1920. Circles represent shell-growth records, and triangles and diamonds represent oxygen and carbon isotope series, respectively. Records with at least 20% of significant combinations are marked with a black star. The color map represents AR(1) trends computed on annually averaged sea surface temperatures from the HadISST1 dataset since 1920. AR(1) time series for bivalve records and temperature grid points were computed using a 50-year sliding window and a 35-year detrending bandwidth. Redness indicates greater loss of stability.

To assess stability changes over recent decades, we measured the strength and direction of trends in both indicators starting from the year 1920. Given that the bivalve series end at different years, the period over which trends are measured varies among records, with end dates ranging between 2000 and 2015. Trend robustness was assessed using the same range of sliding window sizes and detrending bandwidths as in the pre-1920 period. The minimum window size was 35 years, meaning that the earliest indicator values were computed starting in 1954 and progressively later for larger window sizes (see Materials and Methods). Ten records exhibit positive AR(1) trends that are generally robust to different combinations of sliding window size and detrending bandwidth, with more than 75% of the measured trends being positive and median Kendall τ coefficients ranging between 0.38 and 0.85 (Fig. 3A). These records are located in the northwestern North Sea, Hebridean and Irish Seas, northern Norway coast, and off Newfoundland in the northwestern Atlantic (Fig. 3B). Among these, six series exhibit significant trends in 20 to 100% of the tested parameter combinations.

Trends in λ generally mirror those in AR(1) (figs. S4 and S5), but because λ is relatively sensitive to extreme values, it may exhibit more fluctuations over time, resulting in weaker trends when measured with the Kendall τ coefficient (fig. S6). Nevertheless, significant trends in λ add robustness to our findings with AR(1) and ensure that these trends are not driven by changes in the autocorrelation structure of noise (*10*).

### Sensitivity to basin-scale temperature variability

**Fig. 4.**
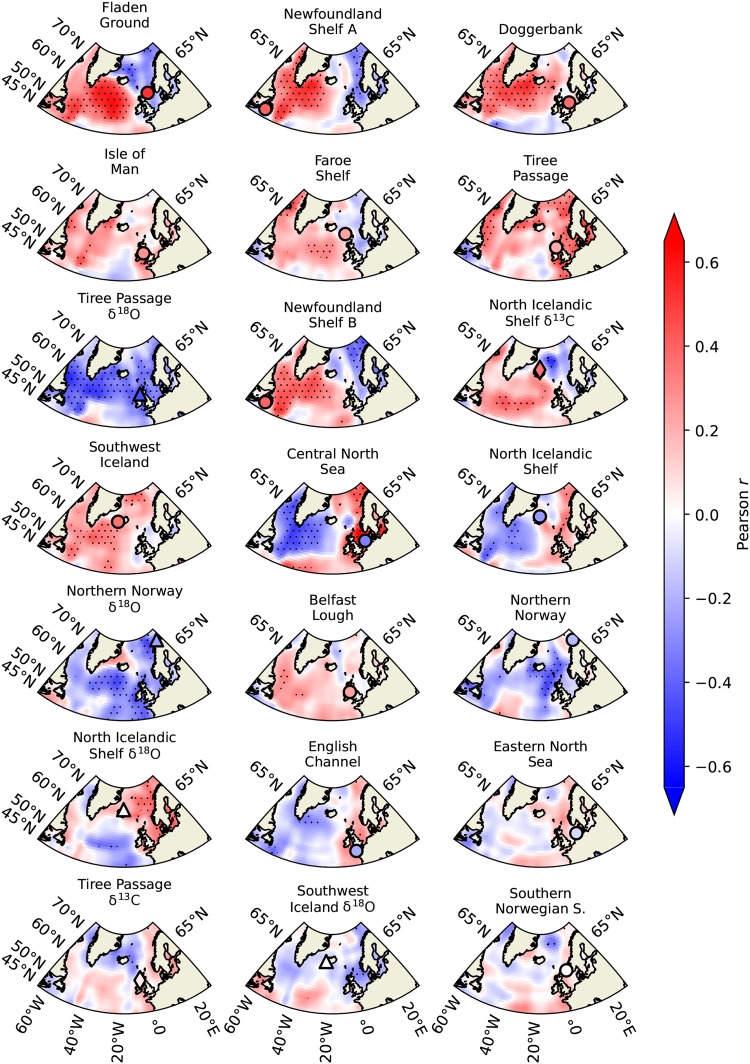
Sensitivity of bivalve records to regional temperature variability. Spatial correlations between each detrended bivalve record and detrended annual near-surface (0 to 100 m) temperatures from the EN4 dataset since 1960. Stippling indicates locations with significant correlations (*P* < 0.1). The location of each record is indicated by a circle, triangle, or diamond corresponding to shell-growth, oxygen, or carbon isotope series, respectively. The color within each geometric figure corresponds to the correlation coefficient between each record and SPG near-surface temperatures (see Materials and Methods).

When comparing changes in AR(1) in the bivalve records with AR(1) trends in surface water temperatures from the HadISST1 (*17*) and EN4 (*53*) datasets (Fig. 3B and fig. S7), we find that the spatial patterns in trends for both bivalve records and temperatures are generally consistent, although variability in the early portion of the EN4 near-surface temperature dataset may partly reflect surface-only data because of limited subsurface observations. The observed slowing down in surface temperature variability is a basin-scale phenomenon occurring over much of the subpolar North Atlantic region (Fig. 3B). To investigate whether changes in variability in bivalve records are linked to basin-scale processes, we calculated spatial correlations between each record and detrended near-surface temperatures since 1960 (see Materials and Methods). Most records exhibit positive correlations with various regions across the subpolar North Atlantic (Fig. 4), indicating their sensitivity to basin-scale variability.

**Fig. 5.**
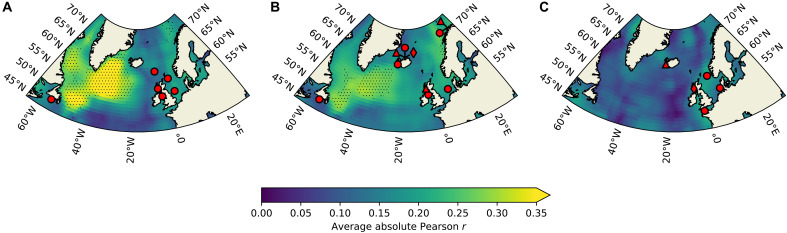
Regions of common sensitivity to temperature variability among bivalve records. Spatial averages of absolute Pearson correlation coefficients between near-surface temperatures from the EN4 dataset since 1960 and (**A**) bivalve records with robust significant AR(1) trends, (**B**) records with weakly positive AR(1) trends (showing more than 50% positive trends in the robustness analysis and a median trend above zero), and (**C**) records with mostly negative trends. Stippling indicates grid points holding significant correlations (*P* < 0.1) with at least half of the assessed records in each case. The scattered figures represent the bivalve records used in each analysis, where circles, triangles, and diamonds correspond to shell-growth, oxygen, and carbon isotope records, respectively.

**Fig. 6.**
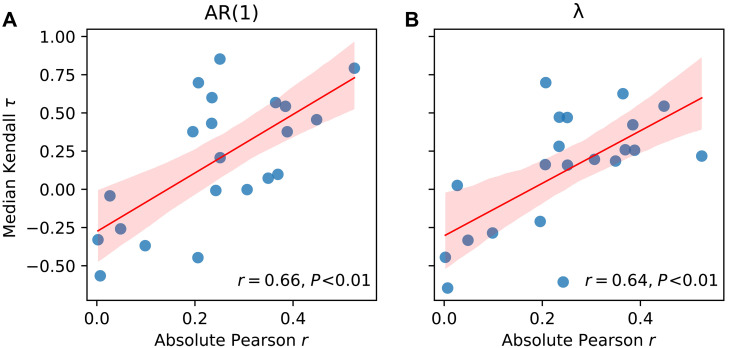
Correlation between SPG temperature sensitivity and the degree of loss of stability recorded in bivalves. Relationship between the absolute correlation of each record with near-surface SPG temperatures since 1960 (*x* axis) and its median (**A**) AR(1) and (**B**) λ trend (*y* axis). Each circle represents a bivalve record, while the solid red line shows the regression trend, and the shaded area represents the associated 95% confidence interval. The strength of the relationship (*r*) is statistically significant in each case, indicating that records with the strongest AR(1) and λ trends are the most sensitive to SPG temperature variability.

To identify regions of common sensitivity among bivalve records, we averaged the absolute correlation coefficients at each grid point for three groups of records: those with significant trends in AR(1), those with weakly positive trends, and those with mostly negative trends (see Materials and Methods) since 1920. This analysis indicates that records exhibiting significant increasing AR(1) trends are particularly sensitive to temperature variability in the SPG region (Labrador and Irminger Seas) and show comparatively lower sensitivity to other regions (Fig. 5A). In contrast, records with relatively weak AR(1) trends demonstrate reduced sensitivity to the SPG area (Fig. 5B). Those with predominantly negative AR(1) trends display weak correlations with basin-scale variability (Fig. 5C). Furthermore, sensitivity to near-surface temperature variability in the SPG is strongly correlated with the degree of stability loss captured by bivalve records (Fig. 6). This suggests that recent critical slowing down in bivalve record variability is a basin-scale phenomenon predominantly occurring in response to change in the SPG region or a common driver.

## DISCUSSION

Because bivalve records integrate multiple environmental signals from local to regional scales (*29*, *41*), attributing the observed slowdown in variability to a specific process is particularly challenging. However, given that physical variability influences all records, and oxygen isotope records reflect exclusively physical environmental variability (*29*, *33*), the results suggest that the observed destabilization may be linked to physical processes. While bivalves are undoubtedly influenced by local conditions, the highly consistent multidecadal AR(1) and λ changes among records from distant locations (Fig. 1), such as Newfoundland, the northern North Sea, Irish Sea, and northern Norway, indicate that this behavior is driven by a basin-scale phenomenon. This is further supported by the fact that the records showing the strongest loss of stability are those most sensitive to regional, particularly SPG, temperature variability (Figs. 5 and 6), in some cases more so than to local temperatures (fig. S8B). The coherent AR(1) and λ changes between bivalve records and SPG temperatures (Fig. 1) lend confidence to earlier stability assessments based on SPG temperatures derived from instrumental data (*10*), confirming that the inferred loss of stability in the SPG region is not an artifact of data aggregation techniques.

The main modes of basin-scale variability in the North Atlantic are the North Atlantic Oscillation (NAO) and the AMV, linked to atmospheric and oceanic processes, respectively, and operating on seasonal to multidecadal timescales (*54*). Although some records show relatively high sensitivity to NAO and AMV indices, there is no significant relationship between the strength of the AR(1) trends and sensitivity to these indices (fig. S8). This suggests that the observed changes in stability indicators are not directly driven by these dominant modes of variability. Instead, SPG temperature and bivalve-inferred variability are likely driven by common dynamics, which may in turn be an oceanic response to atmospheric forcing. Identifying a common driver requires insight into the mechanisms influencing both temperature and bivalve variability. There is an ongoing debate about what drives surface temperature variability in the SPG region. Growing evidence suggests that SPG temperatures do not respond passively to atmospheric forcing as previously thought but that the ocean actively influences its own state (*21*, *55*–*59*), including through feedbacks to the NAO (*60*, *61*). SPG temperature variability is thus shaped by atmospheric dynamics and important contributions from the overturning and SPG circulations on subdecadal to interdecadal timescales (*21*, *55*–*57*). Consistent with this, SPG temperatures are strongly correlated with an altimetry-derived SPG strength index (*r* = −0.85, *P* < 0.01; fig. S9) (*62*), suggesting a link to regional circulation dynamics, although this index may also capture the influence of other processes beyond SPG strength alone (*63*).

Circulation mediates oceanic influences on shelf seas (*64*), which helps explain why records from locations influenced by the subpolar North Atlantic circulation system are highly sensitive to basin-scale variability. The Norwegian Atlantic and European Slope Currents transport a mixture of subpolar and subtropical waters to the Norwegian, Hebridean, and northern North Seas (*43*, *50*, *64*), while the Labrador Current influences the Newfoundland Shelf (*35*). Variability in these currents is strongly modulated by density gradients associated with SPG strength (*64*–*67*). Consistently, among the records showing the largest increases in AR(1), the Newfoundland series is strongly correlated with the SPG strength index (*35*). Moreover, variability in some records is understood through the influence of different water masses modulated by regional circulation processes (*30*, *34*). However, the limited temporal overlap between most bivalve records and direct measurements of SPG and AMOC variability constrains our ability to directly assess the sensitivity of these records to circulation variability. Therefore, while it is not yet possible to attribute bivalve variability to a single driver, the geographic coherence and hydrographic context of the records suggest that regional circulation dynamics are a plausible driver of AR(1) and λ changes, although other drivers such as atmospheric variability may also play a role.

Further supporting the link to ocean circulation dynamics, our results reveal insights not captured in earlier studies: An episode of increasing AR(1) and λ values, indicative of stability loss, is evident between 1800 and 1920, preceding a documented major regime shift in the North Atlantic (*68*). Starting in the 1920s, there was a rapid strengthening of branches of the North Atlantic Current (*68*), followed by shifts in species distributions consistent with an increased influence of Atlantic waters in subpolar regions (*68*–*70*). It has been proposed that changes in SPG strength may have caused this shift (*69*). The observed loss of stability preceding rapid circulation changes is consistent with the behavior of bistable systems approaching a tipping point (*6*, *7*). Moreover, records exhibiting this multidecadal pattern of variability generally exhibit high sensitivity to temperatures in the SPG region (Fig. 4 and fig. S10), although this sensitivity may not have been static over time, particularly for records located near major oceanographic boundaries (*30*). Together, this evidence suggests the possibility that the North Atlantic circulation system, likely the SPG region, lost stability and may have crossed a tipping point in the early 20th century.

The 1920s North Atlantic regime shift coincides with the early 20th century warming, a period marked by rapid global temperature increases (*71*). While changes in global temperatures and the AMV over recent decades are largely attributed to external forcing, such as aerosol and greenhouse gas emissions (*71*, *72*), the earlier warming episode is attributed to a combination of anthropogenic factors and a considerable contribution from internal variability (*71*, *73*, *74*), although the specific mechanisms of internal variability remain debated (*74*). Regional assessments indicate that the northern North Atlantic and surrounding areas experienced the largest temperature anomalies (*71*, *74*), pointing to processes in this region as potential drivers of warming. Rapid changes in North Atlantic circulation (*73*, *75*) and strengthening of the SPG (*76*) are among the proposed mechanisms that support this interpretation. In this context, understanding the consequences of the observed destabilization in the North Atlantic could shed light on the mechanisms driving early 20th century warming.

The pre-1920s destabilization before the documented rapid changes in circulation further suggests the possibility that increasing AR(1) and λ values are associated with circulation dynamics. Circulation and SPG temperatures are influenced by AMOC and SPG strength variability (*21*), each identified as a tipping element at risk of weakening abruptly (*3*). Given that the SPG is a subsystem of the AMOC, disentangling the signals associated with each system remains a key challenge. Moreover, a modeling study has found that the AMOC may undergo intermediate tipping events in response to increased freshwater input before a full collapse (*28*). This scenario, if real, would further complicate attributing observed early warning signals to a specific state within the multistability landscape. The broader impact of SPG destabilization on the AMOC also remains uncertain. While some studies indicate that an abrupt shift in SPG circulation could weaken the AMOC (*4*), others argue that it might have a minimal effect (*5*). This is because a weak SPG would contract, allowing subtropical waters to flow into the eastern subpolar North Atlantic (*5*, *22*), potentially leading to a reorganization of deep convection areas (*5*, *28*). It has been proposed that similar dynamics occurred during Holocene SPG transitions (*26*). However, in a warming world, the projected increase in freshwater input into the subpolar North Atlantic may weaken deep convection across all regions, which has been proposed as a necessary condition for a full AMOC collapse (*5*, *28*).

A further notable feature is that by the late 1990s, AR(1) and λ values reach levels comparable to those observed around the 1920s and have exhibited a nonincreasing trend since (Fig. 1). A similar pattern is evident in AR(1) and λ values for near-surface SPG temperatures (Fig. 1). Concurrently, the SPG has been weakening since the late 1990s, accompanied by reduced convection in the Labrador and Irminger Seas (*77*, *78*), a slowdown of regional ocean circulation (*22*, *58*, *79*–*81*), and increased advection of subtropical waters into the northwestern European Shelf and Nordic Seas (*79*, *81*). These changes coincide with ecosystem shifts consistent with a weakened SPG (*69*) and have been attributed partly to changes in NAO-related forcing (*80*, *81*) and primarily to changes in density gradients (*58*, *79*), which are central to the feedbacks driving SPG bistability (*82*). Moreover, it has been proposed that the observed SPG variability can only be explained if the system is operating near the tipping point, where advective-convective feedbacks self-amplify variability (*83*). Although the weakening trend was briefly interrupted by strong convection between 2014 and 2018 (*77*, *84*), convection shutdown occurred in the Labrador Sea in 2021 and 2023 (*85*), likely triggered by one of the largest freshening events since at least 1960 (*86*). If the observed destabilization reflects circulation dynamics, these events could further destabilize the system and push it closer to a tipping point.

Our results reveal two significant destabilization episodes in the northern North Atlantic since the year 1800. The first occurred before the 1920s regime shift, consistent with the hypothesis that a tipping point may have been crossed in the early 20th century, leading to a reorganization of regional circulation. Understanding this episode may provide insights into North Atlantic dynamics and the mechanisms driving early 20th century climate change. The second episode spans recent decades and is consistent with previous findings of destabilization in a spatial fingerprint derived from reanalysis sea surface temperature data and a proxy-derived reconstruction of AMV (*10*, *12*), supporting the proposition that the region has recently lost stability. Although our methods cannot precisely determine which part of the system is losing stability or how close it is to a tipping point, they suggest that the region is heading toward one. While past destabilization episodes, likely associated with the SPG, did not result in major AMOC changes (*26*, *27*), the current context is different. As we move beyond the Holocene’s safe operating space, increased freshwater input into key convective areas (*24*) raises the risk of reaching critical tipping points in both the AMOC and SPG, with the SPG potentially weakening first.

## MATERIALS AND METHODS

### Selection of bivalve records

We compiled all available bivalve-derived records from the northern North Atlantic and selected them on the basis of their suitability for assessing changes in resilience (table S1). We selected continuous records with at least 100 data points and robust intervals, defined for increment-width series as those where the expressed population signal (a measure of climate signal strength) exceeded 0.85 (*29*, *32*), as reported in the original publications. To minimize the influence of nonclimatic factors on AR(1) and λ calculations, we prioritized versions of increment-width records where the age-related trend was removed using negative exponential detrending, variance was stabilized through ratios, and ARSTAN prewhitening was applied (*32*). We only measured changes in AR(1) and λ, as variance in bivalve records can be susceptible to biases introduced by nonclimatic influences, such as the detrending techniques used to remove age-related trends (*32*). Given that most increment-width records undergo a prewhitening process intended to preserve environmental autocorrelation while removing autocorrelation associated with physiological processes (*29*), the AR(1) magnitudes may vary between records without affecting the trends (*32*). Therefore, when needed for comparison purposes, we calculated residual AR(1) values for each series after removing the mean AR(1) value from the time series.

### Calculation of autocorrelation and λ trends

Time series of AR(1) and λ were computed using a sliding window that moves 1 year at a time over the detrended data. Each indicator value is assigned to the final year of its corresponding window. This approach produces a continuous annual time series for each indicator, shorter than the original by the chosen length of the sliding window. To avoid spurious increases in the indicators caused by underlying trends in the data, we detrended the original series before computing the indicators using a Gaussian kernel function with a prescribed bandwidth (*9*). Small bandwidths preserve high-frequency variability while removing long-term trends and low-frequency variability, whereas larger bandwidths retain more low-frequency signals.

AR(1) quantifies the correlation between each slice of the time series and a lagged version of itself and is computed by fitting a first-order autoregressive model using ordinary least squares (*9*). The AR(1) model is expressed as *x*_*t*+1_ = α**x*_*t*_* + σϵ, where *t* represents time, *x* is the portion of the time series within the window, α is the AR(1) coefficient, and ϵ represents white noise with variance σ^2^.

The restoring rate (λ) quantifies how quickly a system returns to equilibrium after disturbance (*10*). Under the assumption that the system dynamics are approximately linear near equilibrium, they can be represented as d*x*/d*t* ~ λ*x +* η, where *x* is the state variable (i.e., the segment of the time series within a given window), λ is the restoring rate, and η represents high-frequency environmental noise (*10*). The derivative d*x*/d*t* is approximated using the first difference between consecutive observations (*x_t_* − *x_t_*_-1_). λ is then calculated by performing a linear regression of d*x*/d*t* onto *x* within each window. To account for potential changes in the autocorrelation of the noise over time, we applied a generalized least-squares regression with an autoregressive covariance structure (*10*). This approach reduces the risk of false positives that can arise from changes in the noise autocorrelation structure (*10*).

Both stability indicators, AR(1) and λ, were measured only over intervals containing at least 80 data points. Consequently, some records were suitable for assessing either the recent or pre-1920s interval. Consistency for each indicator between records was measured by computing interseries Pearson correlations. To detect the strength and direction of trends in each indicator series, we applied the nonparametric Mann-Kendall test (*9*, *52*). This method evaluates whether there is a consistent upward or downward trend over time by testing for a monotonic relationship between time and the indicator values. The resulting Kendall τ coefficient ranges between −1 (strictly decreasing) and 1 (strictly increasing), reflecting both the direction and strength of the trend. Positive τ values reflect an increase in the indicator over time, suggesting a loss of stability and the potential approach of a tipping point (*9*, *52*).

### Robustness and significance

Because the trend measurement depends on two parameters, window size and filtering bandwidth, we conducted a robustness assessment to ensure that observed trends were not artifacts of specific parameter choices. To assess robustness, we measured the trends across a range of combinations of parameters. We varied the sliding window size from a minimum of 35 years and increased it in increments of 1 year up to a maximum window size equal to 80% of each record size. Filtering bandwidths ranged from 25 years up to 60% of each record length (*52*).

Significance for each combination of parameters was calculated by comparing the trend measured in each record with the expected trends from a null model. Each model consisted of 2000 surrogate series, each generated using a bootstrapping method by sampling with replacement from the residuals of the original time series to ensure that they have the same spectral properties as the original records. We estimated Kendall τ trends for each surrogate series after computing AR(1) using the corresponding window size and bandwidth specific to that parameter combination. The *P* value was defined as the proportion of series that exhibit a τ value greater than or equal to that observed in the original series (*52*).

### Comparison with reanalysis temperature datasets

To compare trends in the bivalve records with observed temperature trends, we used sea surface temperature data from the UK Met Office Hadley Centre HadISST1 reanalysis data and depth-averaged (0 to 100 m) temperature data from the EN4 (EN4.2.2 version) dataset (*17*, *53*). AR(1) trends were computed over the 1920 to 2023 period on the residuals of annually averaged time series from each grid point of the spatial domain in the North Atlantic. A 50-year sliding window and 35-year detrending bandwidth were used to generate the AR(1) time series. To compare trends between the bivalve records and SPG near-surface temperatures, we calculated changes in AR(1) and λ using the regionally averaged SPG temperature series (70°W to 20°W, 45°N to 60°N) from the EN4 dataset, beginning in 1900. AR(1) and λ were computed using a 50-year sliding window after detrending the series with a 15-year Gaussian bandwidth.

To assess the influence of basin-scale environmental variability on bivalve records, we computed spatial correlations between each original series and near-surface temperatures over the northern North Atlantic since 1960 when temperature observations exhibit a considerable uncertainty reduction compared with previous years (*53*). Because bivalves integrate information influenced by processes occurring at different depths that may not be reflected by surface temperatures, the correlations were computed with annual depth-averaged (0 to 100 m) temperatures from the UK Met Office Hadley Centre EN4.2.2 dataset (*53*). This depth range was informed by evidence indicating that the Northwestern European shelves are influenced by oceanic waters from depths between 10 and 100 m (*64*). To avoid spurious correlations arising from long-term trends, we computed Pearson correlation coefficients on the residuals of temperature and bivalve records after removing the trend. The trend was computed using a 35-year moving average, where the weights for the smoothing were determined by a Gaussian kernel function.

To identify the regions in which bivalve records share sensitivity, we averaged the absolute correlation coefficients on each grid point and calculated the proportion of records exhibiting significant correlations with each grid point since 1960. We performed this analysis for three groups of records: (i) those with significantly positive AR(1) trends in at least 20% of combinations in the robustness analysis, (ii) those with weak AR(1) trends (more than 50% positive trends and a median trend above zero), and (iii) those with predominantly negative trends (less than 50% positive trends and a median trend below zero).

To further explore the relationship with SPG temperatures, we computed Pearson correlation coefficients between each record and regionally averaged annual near-surface (0 to 100 m) temperatures in the SPG region since 1960, obtained from the EN4.2.2 dataset (*53*). The SPG region was defined as the 70°W to 20°W and 45°N to 60°N box (*4*). Bivalves and the SPG temperature series were detrended before calculating the correlations. The trend was computed using a 35-year moving average weighted with a Gaussian Kernel function. In addition, we computed Pearson correlation coefficients for the period since 1960 between each bivalve record and detrended local temperatures obtained from the EN4.2.2 dataset, as well as between the bivalve records and the NAO and AMV indices provided by the National Oceanographic and Atmospheric Administration.
