# Supplementary material for: Recent and early 20th century destabilization of the subpolar North Atlantic recorded in bivalves
Source: Sci Adv. 2025 Oct 3;11(40):eadw3468. doi: 10.1126/sciadv.adw3468 (PMC12494007; doi:10.1126/sciadv.adw3468)
Supplement: Supplementary file 1 — Figs. S1 to S10 Table S1 [file sciadv.adw3468_sm.pdf]

Science Advances
AAAS

Supplementary Materials for

Recent and early 20th century destabilization of the subpolar North Atlantic recorded in bivalves

Beatriz Arellano-Nava *et al.*

Corresponding author: Beatriz Arellano-Nava, b.arellano@exeter.ac.uk; Paul R. Halloran, p.halloran@exeter.ac.uk

Sci. Adv. **11**, eadw3468 (2025)
DOI: 10.1126/sciadv.adw3468

This PDF file includes:

Figs. S1 to S10
Table S1

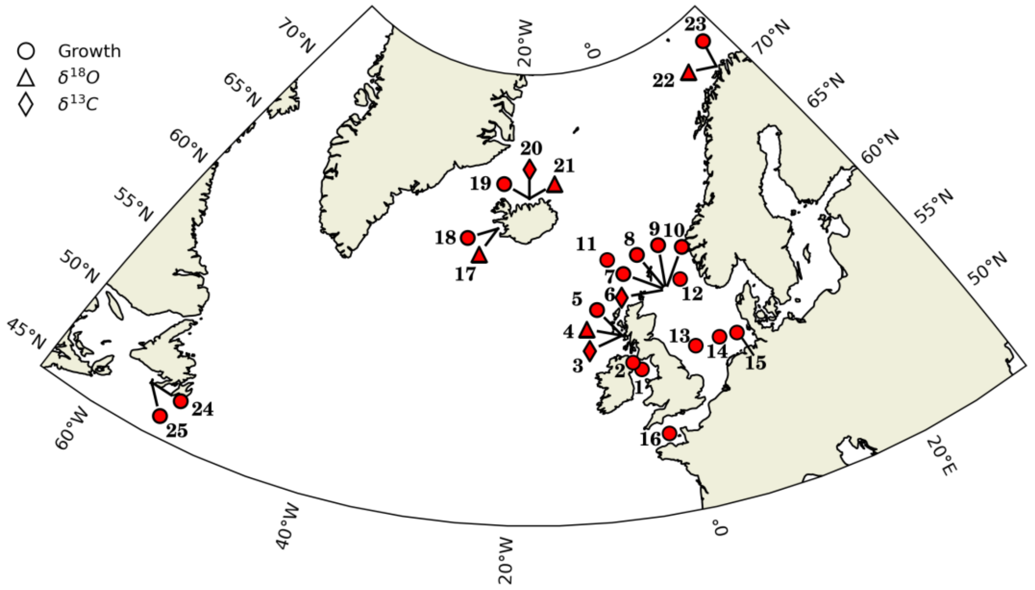

Fig S1. Locations of the bivalve records selected for this study. Circles represent shell-growth records, triangles represent oxygen isotope series, and diamonds represent carbon isotope series. The numbers correspond to the records listed in table S1.

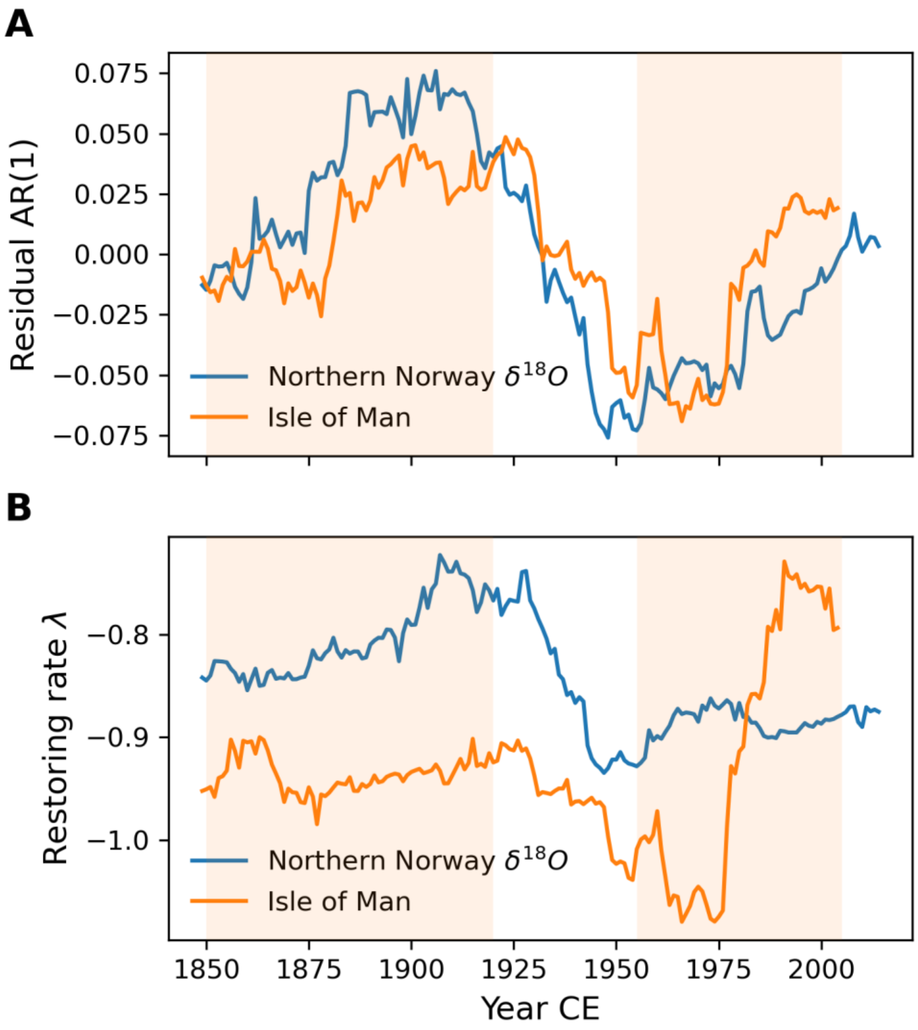

Fig. S2. Changes in AR(1) and λ in two bivalve records with robust data extending back to 1650, both of which exhibit robust and significant trends before 1920 (Figure 2). **(A)** AR(1) and **(B)** λ were computed using a 200-year sliding window after detrending with a 15-year detrending bandwidth.

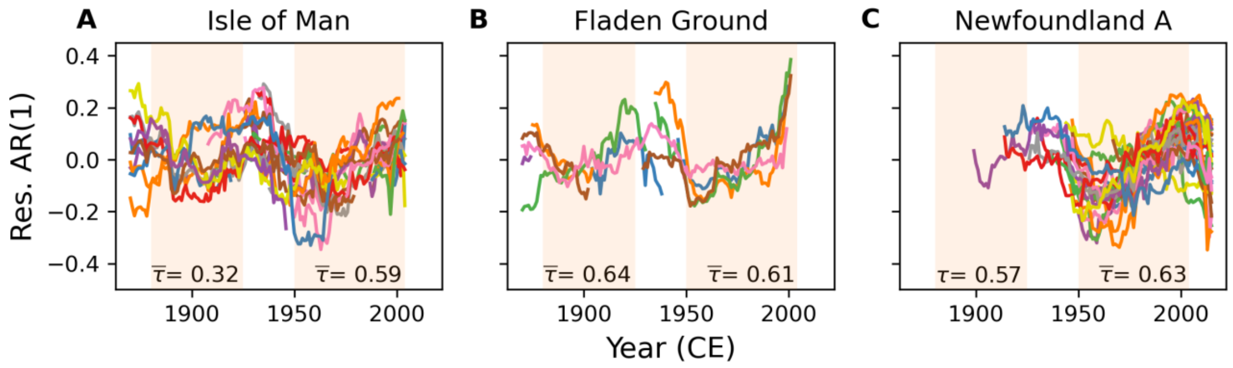

Fig. S3. Temporal evolution of AR(1) for individual shell increment-width series used in the construction of the **(A)** Isle of Man, **(B)** Fladen Ground and **(C)** Newfoundland Shelf A records. These records were selected based on the availability of raw data. Prior to measuring AR(1), the age-related trend was removed using the Negative Exponential method combined with the ratios approach to stabilize variance. AR(1) was computed using a 50-year sliding window with a 15-year detrending bandwidth, with results plotted at the end of each window. Shaded intervals highlight periods of declining resilience. The average Kendall value is shown for each interval and record.

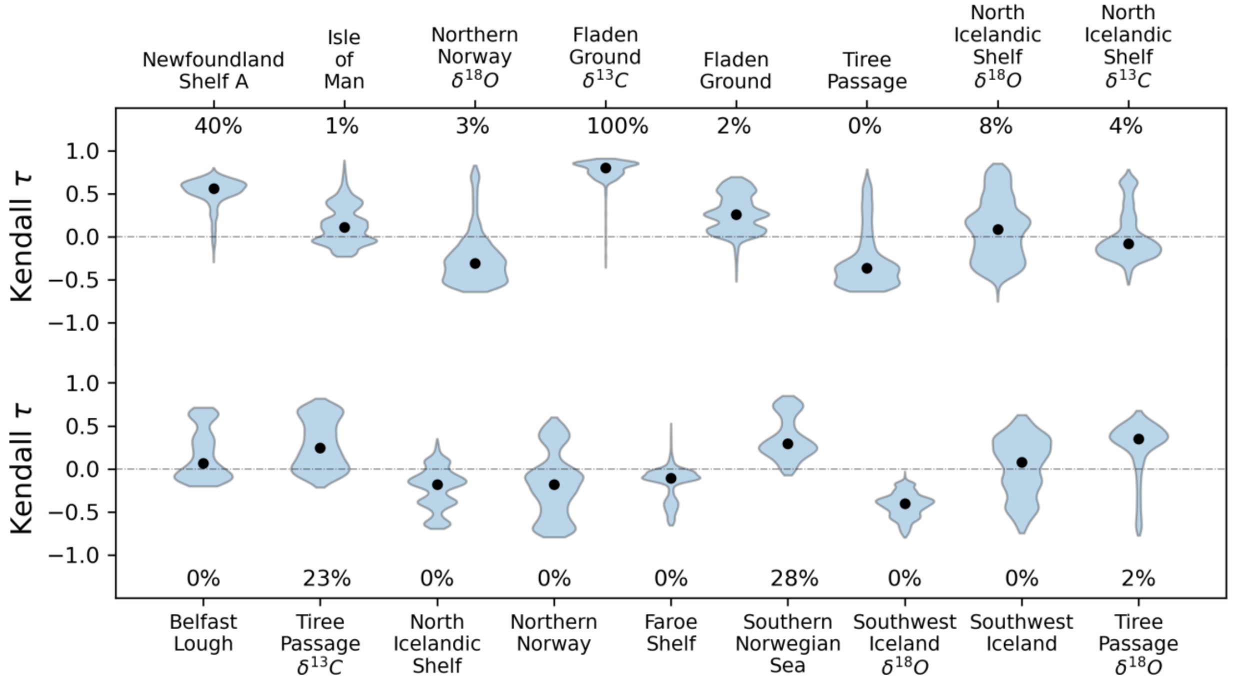

Fig. S4. Robustness and significance of λ trends in bivalve records before 1920. Distribution of Kendall τ trends in λ for all combinations of sliding window and detrending bandwidth measured on each bivalve record between 1750 and 1920. Black circles represent the median Kendall τ values, while the percentages indicate the fraction of combinations exhibiting a significant trend.

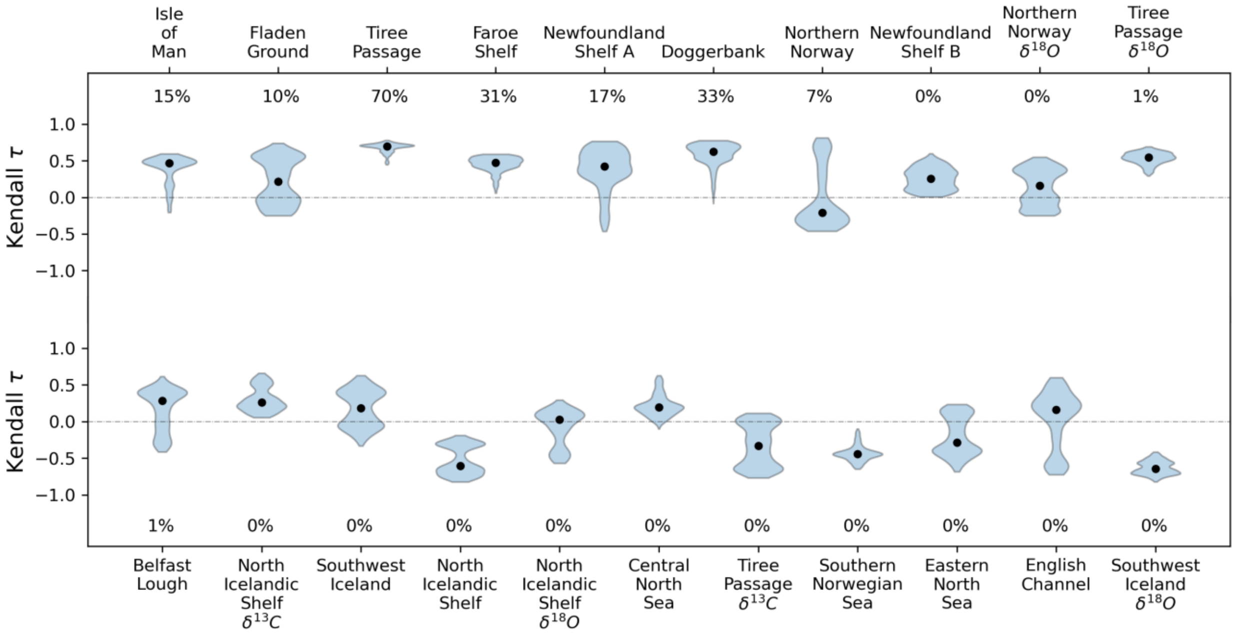

Fig. S5. Robustness and significance of λ trends in bivalve records since 1920. Distribution of Kendall τ trends in λ for all combinations of sliding window and detrending bandwidth measured on each bivalve record since 1920. Black circles represent the median Kendall τ values, while the percentages indicate the fraction of combinations showing a significant trend.

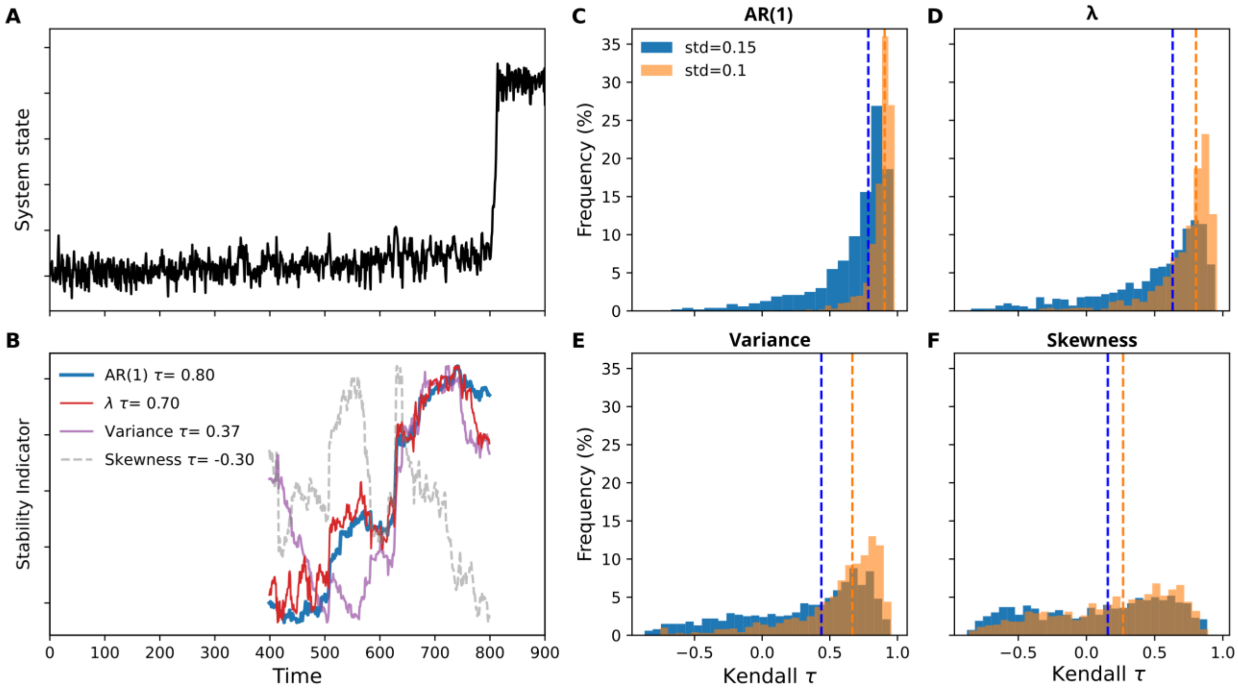

Figure S6. Trends in four stability indicators for a theoretical system gradually approaching a tipping point under two different noise regimes. Panels **(A)** and **(B)** illustrate an example of such a system and the indicator time-series for each one of the proposed stability metrics. Panels **(C)**–**(F)** display the distribution of trends in **(C)** AR(1), **(D)** λ, **(E)** variance and **(F)** skewness, measured using the Kendall coefficient obtained from 5,000 simulated time-series. The vertical dashed line represents the median trend. Each time-series was produced from the stochastic differential equation: $\dot{x}=-x3+x+\mu+\sigma\eta$, where x(t) represents the system state, $\mu=2t\sqrt{3}/(9 \cdot 900)$ is the bifurcation parameter, and ση represents white noise with standard deviation σ set to 0.1 (orange) or 0.15 (blue).

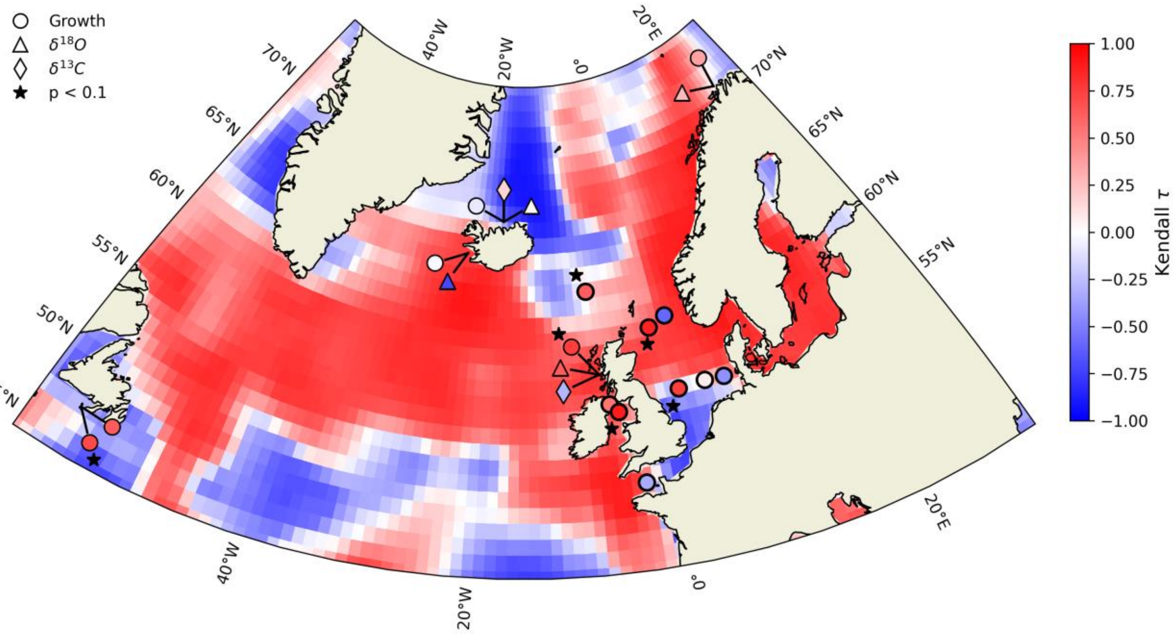

Fig. S7. Spatial trends in AR(1) measured on bivalve-derived records and northern North Atlantic near-surface temperatures. The scattered figures represent AR(1) trends measured on bivalve records since 1920. Circles correspond to shell-growth records, triangles and diamonds to oxygen and carbon isotope series respectively. Records with at least 20% significant combinations are marked with a black star. The colourmap represents AR(1) trends between 1920 and 2022 computed on annually averaged near-surface (0-100m) temperatures from the EN4 dataset. AR(1) time-series for both bivalve records and temperature grid points were computed using a sliding window length of 50 years and a 35-years detrending bandwidth. Trends in these AR(1) time series were then assessed using the Kendall τ test. Redness is associated with the degree of loss of stability.

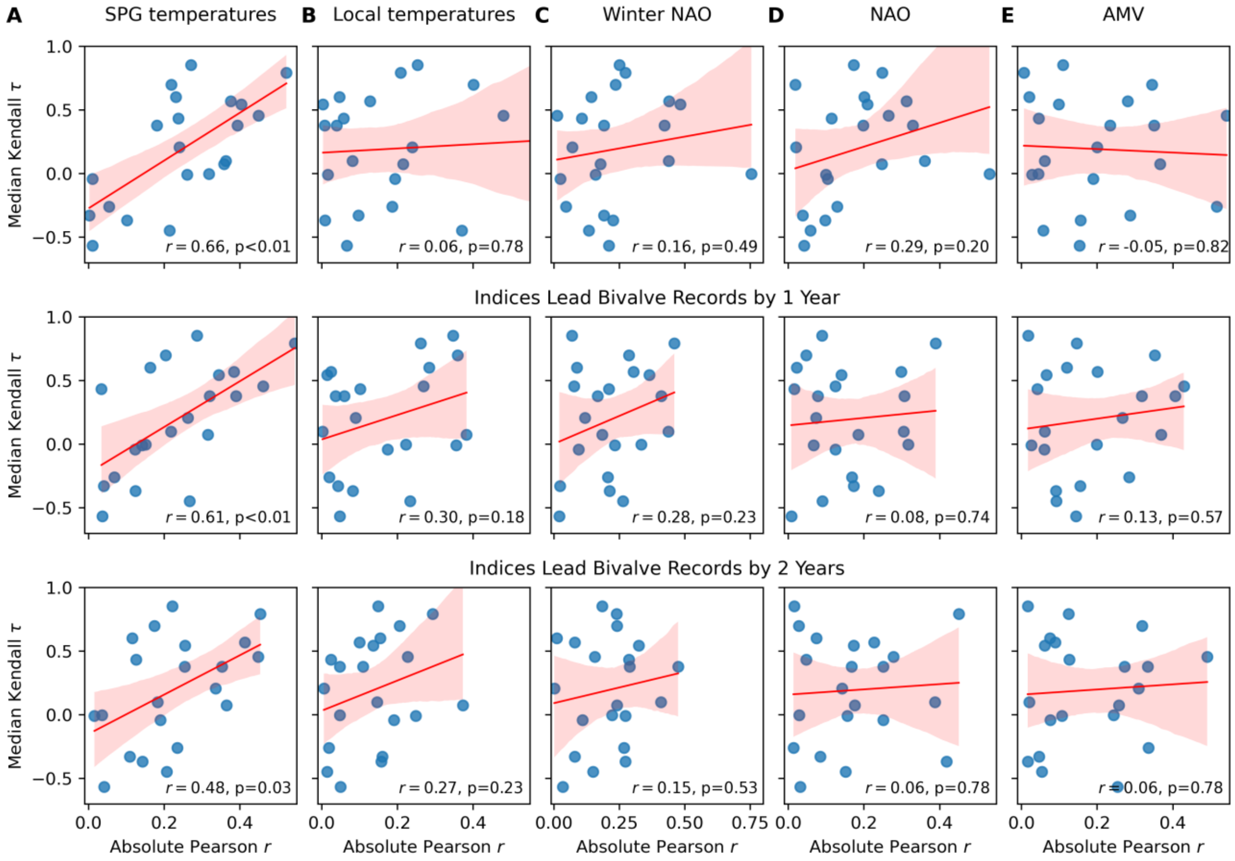

Fig. S8. Correlation between the degree of loss of stability recorded in bivalves and their correlation with main modes of variability and local temperatures since 1960. Relationship between the median AR(1) trend (y-axis) measured in each record and its absolute correlation with **(A)** near-surface SPG temperatures, **(B)** near-surface temperatures in the bivalve record location, **(C)** Winter North Atlantic Oscillation index (averaged for Dec, Jan and Feb), **(D)** annually averaged North Atlantic Oscillation index, and **(E)** Atlantic Multidecadal Variability index. The top row corresponds to relationships during the same year, while the middle and bottom rows correspond to cases where environmental series lead bivalve records by 1 and 2 years, respectively. Each circle represents a bivalve record, while the solid red line shows the regression trend, and the shaded area represents the associated 95% confidence interval. The strength of the relationship (r) along with its p-value is indicated in the bottom right corner of each subplot.

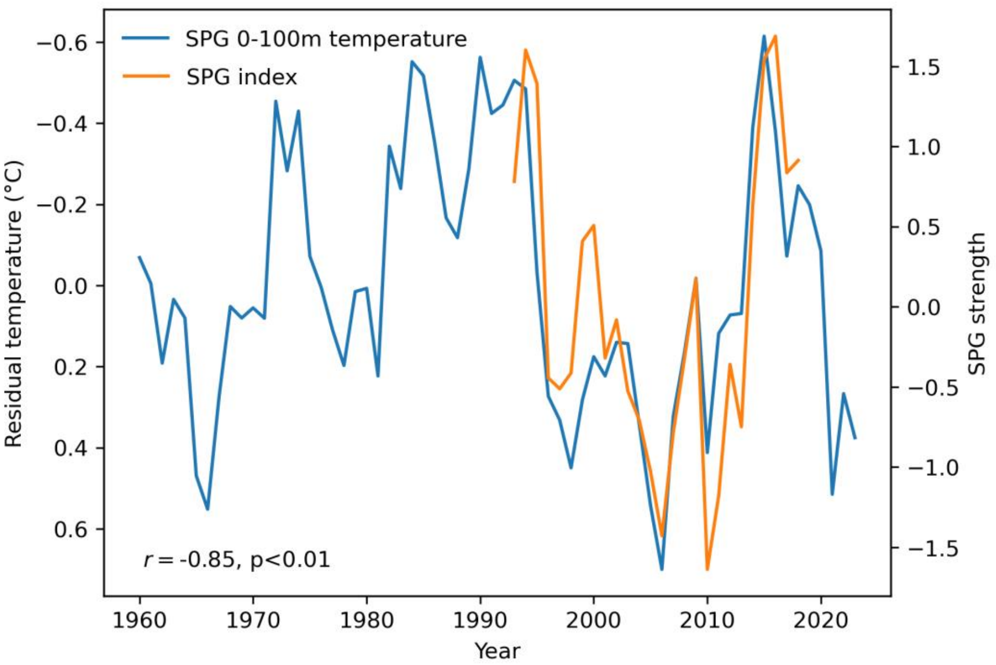

Fig S9. Comparison between an annually averaged altimetry-derived SPG index and residual near-surface (0-100m) EN4 temperatures from the SPG region. Residual temperatures were obtained by detrending with a 35-year moving average weighted using a Gaussian kernel function. The Pearson correlation coefficient between the two series is shown in the bottom left.

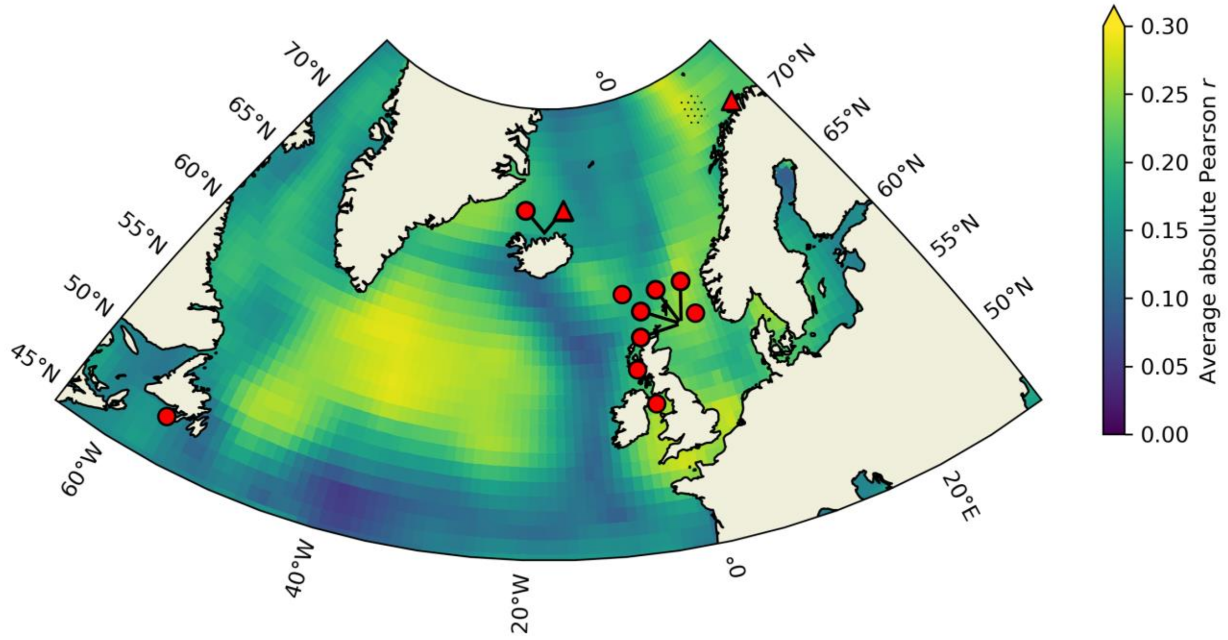

Fig. S10. Regions of common sensitivity to temperature variability. Spatial averages of absolute Pearson correlation coefficients since 1960 between near-surface temperatures and records that exhibit a consistent multidecadal pattern of variability in autocorrelation. The scattered figures correspond to the bivalve records used for each analysis. Circles and triangles correspond to shell-growth and oxygen isotope records, respectively.

Table S1. Details of bivalve records selected for this study and analyzed intervals within each record.

	Record	Type	Location	Species	Robust interval	Examined periods	Reference
1	Isle of Man	Shell growth	Isle of Man, Irish Sea	*A. islandica*	1540-2004	1920-2004<br>1800-2004<br>1750-1920	Butler *et al.* (ref. 40)
2	Belfast Lough	Shell growth	Belfast Lough, Irish Sea	*A. islandica*	1839-2012	1920-2012<br>1839-1920	Román-González & Ridgway in Reynolds *et al.* (ref. 41)
3	Tiree Passage $\delta^{13}C$	Carbon isotope	Tiree Passage, Hebridean Sea	*G. glycymeris*	1799-2010	1920-2010<br>1799-1920	Reynolds *et al.* (ref. 44)
4	Tiree Passage $\delta^{18}O$	Oxygen isotope	Tiree Passage, Hebridean Sea	*G. glycymeris*	1799-2010	1920-2010<br>1799-1920	Reynolds *et al.* (ref. 43)
5	Tiree Passage	Shell growth	Tiree Passage, Hebridean Sea	*G. glycymeris*	1810-2010	1920-2010<br>1810-2010<br>1810-1920	Reynolds *et al.* (ref. 42)
6	Fladen Ground $\delta^{13}C$	Carbon isotope	Fladen Ground, North Sea	*A. islandica*	1551-1915	1750-1915	Estrella-Martínez *et al.* (ref. 36)
7	Fladen Ground	Shell growth	Fladen Ground, North Sea	*A. islandica*	1774-2001	1920-2001<br>1800-2001<br>1774-1920	Estrella-Martínez *et al.* (ref. 36)
8	Fladen Ground B	Shell growth	Fladen Ground, North Sea	*A. islandica*	1870-1979	1870-1979	Butler *et al.* (ref. 37)
9	Fladen Ground C	Shell growth	Fladen Ground, North Sea	*A. islandica*	1870-1979	1870-1979	Butler *et al.* (ref. 37)
10	Fladen Ground F5	Shell growth	Fladen Ground, North Sea	*A. islandica*	1870-1979	1870-1979	Butler *et al.* (ref. 37)
11	Faroe Shelf	Shell growth	Faroe Shelf	*A. islandica*	1648-2014	1920-2014<br>1800-2014<br>1750-1920	Bonitz *et al.* (ref. 46)
12	Southern Norwegian Sea	Shell growth	North-eastern North Sea	*A. islandica*	1820-2002	1920-2002<br>1820-2002<br>1820-1920	Holland *et al.* (ref. 38)

13	Doggerbank	Shell growth	Doggerbank, North Sea	*A. islandica*	1852-2010	1920-2010	Holland *et al.* (ref. 38)
14	Central North Sea	Shell growth	Central North Sea, North Sea	*A. islandica*	1866-2001	1920-2001	Schöne *et al.* (ref. 39)
15	Eastern North Sea	Shell growth	South-eastern North Sea	*A. islandica*	1888-2010	1920-2003	Holland *et al.* (ref. 38)
16	English Channel	Shell growth	English Channel	*G. glycymeris*	1940-2012	1920-2012	Holmes et *al.* (ref. 45)
17	Southwest Iceland $\delta^{18}O$	Oxygen isotope	Southwest Iceland	*A. islandica*	1765-2015	1920-2015<br>1800-2015<br>1750-1920	Mette *et al.* (ref. 49)
18	Southwest Iceland	Shell growth	Southwest Iceland	*A. islandica*	1790-2015	1920-2015<br>1800-2015<br>1750-1920	Mette *et al.* (ref. 49)
19	North Icelandic Shelf	Shell growth	North Icelandic Shelf, Iceland	*A. islandica*	1175-2005	1920-2005<br>1800-2005<br>1750-1920	Butler *et al.* (ref. 47)
20	North Icelandic Shelf $\delta^{13}C$	Carbon isotope	North Icelandic Shelf, Iceland	*A. islandica*	953-2000	1920-2000<br>1800-2000<br>1750-1920	Reynolds *et al.* (ref. 48)
21	North Icelandic Shelf $\delta^{18}O$	Oxygen isotope	North Icelandic Shelf, Iceland	*A. islandica*	953-2000	1920-2000<br>1800-2000<br>1750-1920	Reynolds *et al.* (ref. 33)
22	Northern Norway $\delta^{18}O$	Oxygen isotope	Northern Norway	*A. islandica*	1539-2014	1920-2014<br>1800-2014<br>1750-1920	Mette *et al.* (ref. 50)
23	Northern Norway	Shell growth	Northern Norway	*A. islandica*	1500-2012	1920-2012<br>1800-1920<br>1750-1920	Mette *et al.* (ref. 50)
24	Newfound-land Shelf A	Shell growth	Newfound-land Shelf	*A. islandica*	1850-2015	1920-2015<br>1850-2015<br>1850-1920	Poitevin *et al.* (ref. 35)
25	Newfound-land Shelf B	Shell growth	Newfound-land Shelf	*A. islandica*	1908-2015	1920-2015	Poitevin *et al.* (ref. 51)
